# Hypertensive Emergency Presenting With Isolated Cranial Nerve III Palsy and Subsequent Intracranial Hemorrhage

**DOI:** 10.7759/cureus.99555

**Published:** 2025-12-18

**Authors:** Mohammed Khaleefah, David Parvizi, Huda Nasani, Dylan Begun, Kim Nguyen, Marco Valladres, Huyen Tran, Jhoette Dumlao

**Affiliations:** 1 Family Medicine, Chino Valley Medical Center, Chino, USA; 2 Family Medicine, Chino Valley Medical Center, chino, USA

**Keywords:** brain bleed, family medicine residency, general internal medicine, neurology and critical care, stroke

## Abstract

Hypertensive emergencies present as a life-threatening condition characterized by severely elevated blood pressure with evidence of acute end-organ damage. The most common neurological presentations include ischemic stroke, encephalopathy, and intracranial hemorrhage; however, isolated cranial nerve palsies are a rare occurrence. We present a case of a 72-year-old female with a history of chronic hypertension who presented to the emergency department at Chino Valley Medical Center with a one-week history of malaise, headache, hypertension, and right eye pain. She subsequently developed a right-sided cranial nerve III palsy during hospitalization. CT angiography demonstrated greater than 90% stenosis of the left carotid bulb without evidence of aneurysm. MRI showed bilateral basal ganglia and thalamic T2 hyperintensities consistent with hypertensive encephalopathy. Despite escalating antihypertensive therapy, the patient experienced an abrupt neurological decline and cardiopulmonary arrest. Repeat CT imaging revealed a multi-compartment intracranial hemorrhage with a 10 mm midline shift, with the hemorrhage pattern most consistent with rupture of a previously occult aneurysm not visualized on initial CTA. This case highlights an atypical presentation of hypertensive emergency with an isolated cranial nerve III palsy and underscores the importance of considering aneurysmal pathology even when early vascular imaging is unrevealing. Recognition of such atypical presentations is critical for timely diagnosis and prevention of devastating neurological outcomes.

## Introduction

Hypertensive emergency is defined as a sudden, severe elevation in blood pressure >180/120 mmHg with associated end-organ damage. Commonly affected organs include the retina, kidneys, heart, and brain. With blood pressure elevated past these levels, abrupt vascular endothelial injury develops, leading to increased permeability, fibrinoid necrosis, and microvascular thrombosis. As a result, the renin-angiotensin-aldosterone system becomes activated due to reduced renal perfusion, further worsening ischemia and organ dysfunction [1]. Recent data derived from rat model studies suggest that abnormal angiogenesis, dysfunctional ADAMTS13 activity, and complement activation may contribute to microvascular and thrombotic injury in hypertensive emergency [2].

Hypertensive emergency is distinguished from hypertensive urgency by the presence of acute end-organ injury, whereas hypertensive urgency lacks such involvement and is typically managed conservatively [3-6].

Hypertensive emergency can manifest with a wide range of neurological, renal, and cardiovascular findings. Patients may present with confusion, seizures, encephalopathy, or acute kidney injury, and these organ injuries may appear together or in isolation [7]. Cardiac manifestations such as left ventricular dysfunction and heart failure have also been reported, sometimes even in the absence of symptoms [4]. Presentations frequently include pulmonary edema, ischemic stroke, chest pain from acute coronary syndrome, altered mental status, or symptoms of aortic dissection, with pulmonary edema and ischemic stroke being the most common [8]. Laboratory findings may reveal elevated creatinine, proteinuria, or evidence of microangiopathic hemolytic anemia. Hypertension-related symptoms such as headache or dizziness may also occur, while precipitating factors often include medication noncompliance, dietary sodium excess, sympathomimetic exposure, renal disease, or endocrine abnormalities [8].

Angiotensin-converting enzyme (ACE) inhibitors or angiotensin II receptor blockers (ARB) are commonly used and initiated cautiously with gradual titration, as this strategy has been associated with improved long-term outcomes [9]. Additional management varies depending on the organ involved, such as the use of specific agents in heart failure, aortic dissection, or stroke [10]. In contrast, hypertensive urgency is generally managed in an outpatient setting with oral antihypertensives, and rapid intravenous reduction is not recommended [11].

Hypertensive emergency historically carried high mortality due to acute and chronic organ injury. Five-year survival rates were once below 25%, but improvements in blood pressure management have increased survival to 74% [12]. Short-term morbidity and mortality vary widely depending on the organ system involved, with neurovascular complications carrying the highest early mortality. Although hypertensive urgency has a more favorable prognosis, patients remain at increased long-term risk for cardiovascular events compared to normotensive individuals [13].

## Case presentation

This is a report of a 72-year-old female with a past medical history of hypertension and right-sided retinal artery occlusion who presented to the emergency department (ED) at Chino Valley Medical Center with a one-day history of a severe headache located posterior to her right eye. The patient was feeling unwell earlier that day, went to take a nap, and was woken up due to the pain. That day, she took hydralazine 25 mg, carvedilol 12.5 mg, and 0.2 mg of clonidine after she began having her symptoms. She denied any nausea, vomiting, recent illness, or other complaints. Her vitals showed she was hypertensive at 199/74 with all other vitals normal. Her admission labs showed BUN 19 mg/dL and creatinine 1.9 mg/dL, consistent with acute kidney injury relative to baseline. Her chest X-ray and CT head without contrast were both normal in the ED.

She was then admitted for a hypertensive emergency and acute kidney injury. In the ED, she was given morphine injection 2 mg IV, Benadryl injection 50 mg, Reglan injection 5 mg, hydralazine injection 5 mg, potassium chloride 20 mEq tablet, Norco 5-325 mg tablet, and Catapres 0.1 mg tablet.

The patient was admitted and started on amlodipine 5 mg, losartan 50 mg, nifedipine 30 mg, and hydralazine 25 mg three times a day for oral blood pressure control. She was also given intravenous pushes of both hydralazine 10 mg IV PRN and labetalol 10-20 mg IV PRN for blood pressures greater than 160 systolic.

The patient's symptoms persisted, and her blood pressure remained uncontrolled despite these measures, so medications were titrated up through the hospital stay. Neurology was consulted due to her continued headache in the setting of hypertensive emergency and AKI. Neurology advised that if hypertension was controlled but the headache persisted, then an MRI would be warranted. They recommended neuro checks every four hours as well as repeating the CT for any acute neurological changes. Nephrology was also consulted, recommending a 2 g sodium diet as well as uptitration of blood pressure medications as needed. At this time, blood pressure had come down into the 150-180 range; however, the patient's headache persisted.

On 08/02/2025, the patient developed new right-sided ptosis and impaired adduction of the right eye, consistent with a cranial nerve III palsy on physical examination, and her headache persisted despite continued uptitration to maximal doses of oral and PRN IV medications. On 08/03/2025, further imaging was obtained due to ongoing symptoms. Repeat CT head without contrast, MRI orbit, and CT venogram were unremarkable. CT angiography found >90% stenosis of the left carotid bulb at the bifurcation. Aspirin and Plavix were started immediately; however, the CTA head did not show any aneurysms greater than 3 mm.

On 08/04/2025, MRI brain without contrast showed abnormal T2 hyperintensity in the bilateral basal ganglia and thalami.

On 08/05/2025, the patient's blood pressure continued to fluctuate and rose to 225/85 despite being on four oral and two IV PRN antihypertensive medications. At this point, she was transferred to the ICU for nicardipine drip initiation and underwent another MRI of the brain with contrast, which did not show any abnormal enhancement on post-contrast imaging.

Several hours after the MRI study, the patient was found obtunded, non-responsive, and went into cardiac arrest. One round of cardiopulmonary resuscitation (CPR) achieved return of spontaneous circulation, and she was intubated for airway protection. She remained in the ICU for ventilatory support and blood pressure management.

Post-code assessment revealed both pupils were blown and unreactive. A new post-code CT head (Images) without contrast showed a multicompartment acute intracranial hemorrhage with a right-to-left midline shift of 10 mm. Although the initial CTA did not identify an aneurysm, the hemorrhage pattern-multi-compartmental with significant mass effect-was most consistent with rupture of an underlying intracranial aneurysm not previously visualized.

Hypertonic saline and mannitol were started. Nicardipine drip was continued with a target systolic blood pressure of 160-180 per neurology recommendations. The patient was then transferred to Arrowhead Regional Medical Center for further neurosurgical evaluation.

**Figure 1:**
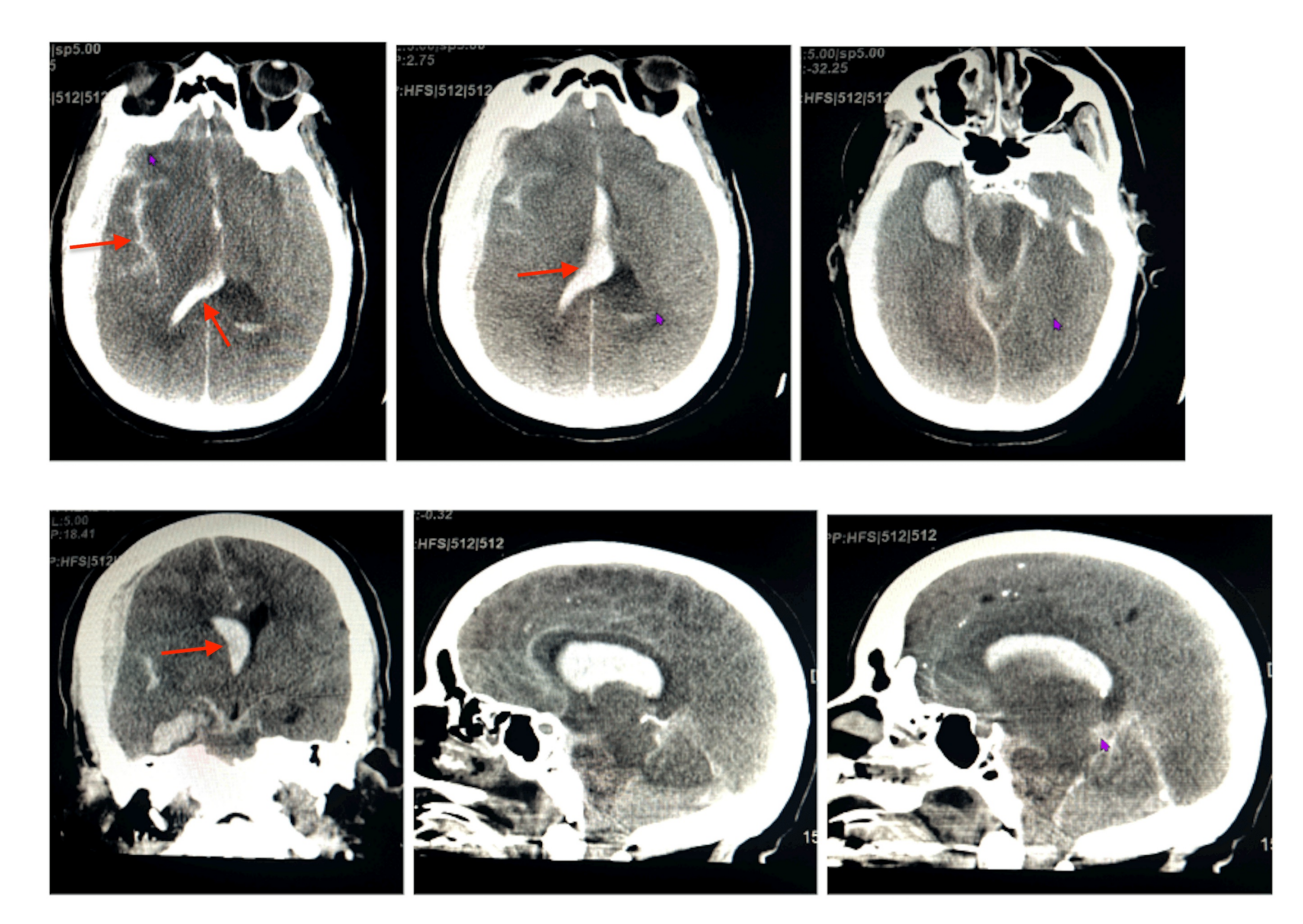
CT head without contrast showing massive hemorrhage

## Discussion

This patient presented to the emergency department with an intractable unilateral headache, hypertensive emergency, and right eye pain. Over the course of three days, despite anti-hypertensive management, she developed a cranial nerve three palsy likely secondary to acute enlargement of an underlying berry aneurysm and eventually a massive intracranial hemorrhage. This case underscores how a seemingly isolated hypertensive headache can mask multi-system hypertensive end-organ damage, which includes neurological, renal, and vascular compromise. The brain imaging for this patient, both on admission and after her CN3 palsy, was read without any acute abnormality. Just hours later, the patient developed a multicompartment intracranial hemorrhage, which highlights the unpredictable nature of malignant hypertension. Moreover, this patient's hypertensive emergency caused an isolated cranial nerve III palsy, which is an extremely rare complication. The most common neurological consequences of hypertensive emergencies are acute ischemic stroke, cerebral hemorrhage, and hypertensive encephalopathy [14]. The vast majority of isolated cranial nerve III palsies occur in diabetes or microvascular ischemia, not an acute blood pressure crisis [15]. This constellation of findings is notable because it highlights the importance of considering and rapidly excluding any emergent pathologies.

Hypertensive emergencies are a well-established cause of acute neurologic injury, including intracerebral and subarachnoid hemorrhage, and can also precipitate vascular events such as carotid dissection or ischemia. However, isolated cranial nerve III palsy is an extremely rare complication. The American College of Cardiology and American Heart Association emphasize that hypertensive emergencies most commonly present with acute heart failure, stroke, or acute kidney injury [14]. In our case, the patient presented to the emergency department with a hypertensive emergency, unilateral right-sided headache, and eventually developed a cranial nerve three palsy likely due to ischemic neuritis or direct mass effect of the aneurysm. This case shares several features that are quite common consequences of hypertensive emergencies, such as kidney injury and headache; however, the reported cases of cranial nerve three palsies are few [15]. It is also important to recognize that any sort of severe end-organ damage, especially focal neurological deficits, should prompt more aggressive blood pressure medications as well as prompt transfer to the critical care unit.

## Conclusions

This case illustrates an uncommon and devastating progression of hypertensive emergency that began with what appeared to be a benign, localized headache and evolved into isolated oculomotor nerve palsy, carotid bulb stenosis, and ultimately intracranial hemorrhage. Although hypertension is a familiar clinical entity, its capacity to produce such focal neurologic and vascular complications underscores how unpredictable and aggressive malignant hypertension can be when autoregulation fails. The initial absence of neuroimaging findings served as a false reassurance, reminding clinicians that early imaging may lag behind ongoing vascular injury.

Our patient's rapid decline highlights the importance of early recognition and a comprehensive, multidisciplinary approach to hypertensive crises. Even subtle or isolated neurological findings should prompt urgent investigation for evolving end-organ damage, particularly when accompanied by refractory elevations in blood pressure. This report reinforces the need for vigilance in monitoring and titrating therapy, as well as for continued research into the mechanisms linking severe hypertension with acute vascular events. In sum, this case serves as a sobering reminder that malignant hypertension, though clinically heterogeneous, demands timely and aggressive management to prevent irreversible outcomes.
